# Evaluation and Treatment of Congenital Syphilis: A National Survey of US Pediatric Specialists

**DOI:** 10.3390/jcm13206280

**Published:** 2024-10-21

**Authors:** David B. Banks, John M. Flores, Jose Luis Paredes, Simon L. Parzen-Johnson

**Affiliations:** 1 The University of Chicago Pritzker School of Medicine, Chicago, Illinois, IL 60637, USA; simon.parzen-johnson@bsd.uchicago.edu; 2 Department of Pediatrics, Section of Pediatric Infectious Diseases, Comer Children’s Hospital, The University of Chicago, Chicago, Illinois, IL 60637, USA; john.flores@uchicagomedicine.org; 3 Department of Medicine, Section of Global Health & Infectious Diseases, Comer Children’s Hospital, The University of Chicago, Chicago, Illinois, IL 60637, USA; 4 Department of Medicine, Advocate Illinois Masonic Medical Center, Chicago, Illinois, IL 60657, USA; jlparedessosa@gmail.com

**Keywords:** congenital syphilis, infectious diseases, pediatrics

## Abstract

**Background/Objectives**: As congenital syphilis incidence continues to increase yearly in the United States (US), recommendations from government and professional organizations aim to inform effective clinical practice, although it is unclear how closely these recommendations are followed. This study surveyed US pediatric specialists regarding their approach to congenital syphilis diagnosis and treatment to examine decision-making relative to practice guidelines and subspecialty. **Methods**: US pediatric physicians recruited from subspecialty directories were sent an online survey conducted in March–April 2024. The case-based survey elicited diagnostic and treatment decisions for different case definitions of congenital syphilis (proven or highly probable, possible, and less likely). **Results**: Among 442 respondents (56.8% women, 74.2% age 40–69, 57.7% 15+ years since training completion), 94.1% chose to evaluate and manage proven or highly probable congenital syphilis as recommended whereas only 45.8% did so for congenital syphilis considered less likely. Diagnostic and treatment decisions by infectious disease specialists and other subspecialists differed across case definitions. **Conclusions**: Physicians’ approaches to congenital syphilis workup and management, including the decision to treat, varied with case presentation where decision-making seemed to diverge from published recommendations and between subspecialists as infection became less likely by case definition.

## 1. Introduction

Congenital syphilis is a systemic infectious disease of the newborn caused by vertical transmission of *Treponema pallidum* from the birthing parent to infant either in utero or at the time of delivery [1]. Despite the continued sensitivity of *T. pallidum* to penicillin, where the risk of congenital syphilis infection and stillbirth are reduced to 97% and 82% when pregnant patients with syphilis receive timely treatment with penicillin, and calls to bolster preventative maternal testing and treatment, the incidence of congenital syphilis has increased over the last decade alongside rates of syphilis in persons of childbearing age in the United States (US) [2,3]. A total of 3761 cases of congenital syphilis were reported to the US Centers for Disease Control and Prevention (CDC) in 2022 alone, a 37.1% increase from 2021 and more than ten times the number of congenital syphilis infections recorded in 2012 [3]. These data showed that lack of timely testing and adequate treatment during pregnancy contributed to 88% of cases of congenital syphilis, and these gaps present in the majority of cases across all races, ethnicities, and geographic regions in the US [3]. Missed or inadequately treated infection can result in short-term health sequelae for the infant including dermatologic conditions, ocular impairment, hydrops fetalis, pneumonia, central nervous system diseases, hepatitis, anemia, thrombocytopenia, stillbirth, and postnatal death [1,4,5]. Additionally, there may be long-term health sequelae including musculoskeletal abnormalities, hearing loss, vision loss, stunted growth, cardiac abnormalities, morbid dermatologic manifestations, and neurodevelopmental delay. To prevent these conditions, early detection and treatment of newborns with congenital syphilis is critical as incidence climbs [1,4,5].

The CDC and American Academy of Pediatrics Committee on Infectious Diseases both publish guidelines for congenital syphilis diagnosis and treatment [1,6]. How closely these recommendations are followed is an ongoing topic of study. One large survey-based study involving neonatologists described differences in diagnosis and management among this one specific subspecialty [7]. An additional local survey study of primary care and specialist pediatric providers in Chicago, Illinois, also demonstrated differences in diagnosis and management practices of congenital syphilis that varied with CDC case definition, while a retrospective cohort study drawing data from 54 major tertiary children’s hospitals in the US noted clinical heterogeneity of diagnosis and management practices related to congenital syphilis using electronic health record data [1,8,9]. 

While some evidence exists for variability in practice, few nationwide US studies engage physician decision-making directly. To this end, we surveyed US pediatric providers regarding their approach to evaluating and treating congenital syphilis and report their decision-making relative to available guidelines and specialty. The primary objectives were to assess the perspectives of congenital syphilis among pediatric providers in terms of diagnosis and management practices related to the various case definitions of congenital syphilis based on published guidelines [6]. Additionally, we sought to investigate if there were significant differences between pediatric infectious disease specialists and non-infectious disease specialists.

## 2. Materials and Methods

We conducted a cross-sectional national survey of pediatric providers in the US regarding their approach to congenital syphilis infection in terms of both diagnosis and treatment. This research study was reviewed and deemed exempt by the Institutional Review Board of the University of Chicago Biological Sciences Division (IRB24-0424). The survey was developed by the research team and included questions about current practice, diagnostic and treatment choices in response to short clinical vignettes based on CDC congenital syphilis case definitions [6], and demographic attributes (Appendix A: Survey instrument questions). Inclusion criteria for this study included board-certified general pediatric and/or pediatric subspecialty medical doctors, nurse practitioners, physician assistants, and advanced practiced providers, those who were actively in practice, practiced in the US, and had English proficiency to complete the survey. 

The first survey question identified healthcare practitioners involved in the management of congenital syphilis in their current practice, which was used to define eligible survey responses, while the second characterized practice as a medical doctor (MD or DO), nurse practitioner (NP or APN), physician assistant (PA), or advanced practitioner (APP) in pediatric infectious diseases, neonatal–perinatal medicine, general pediatrics, and/or pediatric hospital medicine with the option to specify some other combination of credentials and specialization. 

The survey then queried diagnostic and treatment decisions for clinical scenarios corresponding to different case definitions of proven or highly probable, possible, and less likely congenital syphilis by AAP and CDC guidelines outlined in Table S1. Clinical vignettes presented the following case-defining variables and were followed by an explicit case definition including maternal infection and treatment history, maternal and newborn serum nontreponemal (RPR, reactive plasma reagin) titers, and any presenting signs and symptoms in the newborn. Based on the vignette and case definition, respondents could then select any combination of the five diagnostic study choices provided or opt for no additional workup. For treatment, choices included one of three penicillin regimens, no treatment, and to forgo a decision to wait for diagnostic study results. The final four survey questions pertained to basic demographic information including the age of the respondent, years since completion of the terminal training, gender identification, and the geographic region of practice within the US (West, Midwest, South, Northeast) with an accompanying map having color-coded identifications.

The survey was conducted in March and April 2024 via Qualtrics and distributed to 4214 pediatric infectious disease (ID) and neonatal–perinatal medicine physicians and advanced practice providers identified from their respective AAP member listings, as has been the case in previous studies [7,10]. Through email communication, with the first email sent on 1 March 2024, providers were sent an introduction to the research study including its purpose and a link to the survey. This was followed by four weekly reminders, the last of which was sent on the final day of study, and responses were gathered and de-identified prior to data analysis. We ceased acceptance of surveys on 30 April 2024, which was approximately 4 weeks after the final reminder email. Only eligible responses defined by responding “yes” to the first survey question were analyzed. Participation in the study was voluntary with no financial or other secondary incentives offered. Descriptive statistics were used to characterize survey respondents in terms of specialty and queried demographics and responses from pediatric ID and non-ID specialists were further compared using chi-squared (χ^2^) analyses where statistical significance was set at a *p*-value less than 0.05. 

## 3. Results

A total of 544 responses were received, of which 442 (81.3% of 544) were from eligible respondents who completed at least 10 of the 12 survey questions for an effective return rate of 10.7% (442/4124). Respondent demographics are summarized in Table 1. A majority of the survey respondents were women (251/442, 56.8%) between 40 and 69 years of age (328/442, 74.2%) who had completed training 15 or more years ago (255/442, 57.7%). While no majority was defined by region of practice, the US South was most represented overall (175/442, 39.6%). ID (153/442, 34.6%) and non-ID (289/442, 65.4%) specialists differed significantly in age (*p* = 0.008), years since training completion (*p* < 0.001), and region of practice (*p* = 0.013) but not gender, where ID physicians were relatively younger, sightly further past training completion, and more often practiced in the Midwest relative to non-ID respondents. Differences with regard to actions taken relative to recommendations outlined in Supplemental Table S1 for cases of proven or highly probable, possible, and less likely congenital syphilis are described in Table 2, Table 3 and Table 4 [6].

For proven or highly probable congenital syphilis, all respondents pursued some form of additional workup while most (414/442, 94.1%) opted to treat with the preferred or alternative regimen of penicillin without delay, as recommended (Table 2). Some diagnostic tests were favored differently by ID and non-ID clinicians, where 8% fewer ID (139/153, 90.8%) than non-ID (287/289, 99.3%) physicians included long-bone radiography in their evaluation (*p* = 0.003). While the CDC recommends long-bone radiography for all cases of proven or highly probable congenital syphilis, AAP guidelines differ slightly by recommending it as clinically indicated rather than for all cases. While the same is true for both guidelines with respect to other tests beyond blood and cerebrospinal fluid (CSF) studies, 47.5% (210/442) and 82.4% (364/442) of all respondents opted for head imaging and ophthalmologic examination, respectively.

For possible congenital syphilis, treatment approach rather than diagnostic test selection differed between groups (Table 3). ID physicians (35/153, 23%) more often chose to wait for test results before making treatment decisions compared to other specialists (37/289, 13%; *p* = 0.034), a measure the CDC recommends before qualifying single-dose benzathine penicillin G therapy as an option over the preferred 10-day regimens when congenital syphilis is possible. In this regard, 10% of non-ID (30/289) and 5% of ID (8/153) physicians chose to give single-dose benzathine penicillin G without considering any diagnostic findings. While guidelines suggest clinicians may forgo additional workup altogether for possible congenital syphilis if a 10-day regimen is completed, only 3 of the 17 respondents (18%) who opted to forgo workup would ultimately select that regimen. 

When congenital syphilis was less likely, more ID (121/145, 83.4%) than non-ID (198/276, 71.7%) clinicians chose to forgo additional workup (*p* = 0.008) and opted to treat with single-dose benzathine penicillin G (108/153, 70.6%) compared to other physicians (137/285, 48.1%; *p* < 0.001), while non-ID specialists (126/285, 44.2%) more often declined to treat at all relative to those in ID (36/153, 24%; Table 4). Overall, fewer than half of all respondents (193/421, 45.8%) chose to treat with single-dose benzathine penicillin G without additional workup as recommended for less likely congenital syphilis while 28.2% (119/421) would forgo workup and treatment altogether. 

## 4. Discussion

We report differences between infectious diseases and other pediatric specialists in their workup and management of congenital syphilis, including the decision to treat, which varied with the likelihood of infection or case definition at presentation. Decisions made by respondents were most uniform and consistent with guideline recommendations when congenital syphilis was proven or highly probable relative to cases of possible or less likely congenital syphilis. In contrast, the greatest differences in decision-making between specialists, and of both groups relative to published recommendations, were observed when congenital syphilis was less likely but not yet ruled out. Pediatric infectious disease providers tended to wait for the results of diagnostic workup before making management decisions compared to non-infectious disease pediatric providers. Additionally, pediatric infectious disease providers were less inclined to pursue lumbar punctures for possible or less likely congenital syphilis cases, despite CDC guideline recommendations. Taken together, these results suggest variations in clinical practice may increase as presenting congenital syphilis infection likelihood decreases or diagnosis becomes more nuanced or ambiguous. 

The clinical vignettes for this study were formulated to assess management strategies, whereas true patient scenarios are more complicated and exist in less controlled environments. Our survey is unable to capture these conditions, further highlighting the importance of well-designed guidance despite being a limitation of our work. Additionally, demographic differences between infectious disease and non-infectious disease physicians may confound some of our analyses, which we performed in terms of specialty as a single variable of interest for this report. In terms of practice region, for example, decision-making may involve some appraisal of pretest probability that depends on local disease prevalence which varies across the US [11].

The diagnosis of congenital syphilis may be clinically challenging. The 2021 CDC STI Guidelines note that congenital syphilis can be classified in four distinct categories or scenarios, (1) symptomatic or proven; (2) possible; (3) less likely; and (4) unlikely, which affects the treatment course [6]. However, diagnosis of congenital syphilis can be difficult because maternal nontreponemal (RPR) and treponemal immunoglobulin G (IgG) antibodies can be transferred through the placenta to the fetus, complicating the interpretation of reactive serologic tests for syphilis among infants who are less than 30 days of age, which may not necessarily warrant treatment or diagnosis [12,13]. Additionally, nationwide cases of congenial syphilis may be dependent on reporting practices of infant RPRs to departments of public health and then to the CDC [14]. Further contributing to the complexity in diagnosing and treating congenital syphilis, the most utilized diagnosis coding system—the International Classification of Diseases (ICD)—does not have these CDC diagnostic definitions or reported surveillance as codes but rather employs more broad terms such as “Congenital syphilis, unspecified” (ICD-10-CM A50.9) as well as symptom-specific codes such as “Early congenital syphilitic pneumonia”(ICD-10-CM-A50.04s) [15]. As the process of creating a CDC-defined clinical diagnosis (symptomatic or proven, possible, etc.) is translated into a discordant ICD-10 billing code by the provider [16], and then reported to departments of public health who must create a different CDC-recommended surveillance definition of probable or confirmed, there are multiple steps in the process where errors and misclassifications may occur. 

Adding to this complicated process, there are scenarios where the infant may be asymptomatic but still warrant a diagnosis of congenital syphilis with penicillin treatment depending on the adequacy of maternal treatment and/or maternal and nontreponemal serology results, even in the setting of a negative infant RPR. In certain contexts, these infants are diagnosed and treated for congenital syphilis but not reported to local or national public databases [6,13]. These various barriers may lead to significant heterogeneity in diagnosing, managing, and reporting this increasingly prevalent disease, which further reinforces the importance of the findings in our study and the potential for using pharmaceutical administration data to assist in accurately identifying the extent of the current epidemic.

Additional studies looking into other primary acquired sexually transmitted infections [17,18] and sexual assault cases [19,20] have also found discrepancies between surveillance case definitions, guidelines, and clinical utilization among providers, which may lead to heterogeneous practices and reporting, as well as exclusion of true cases to affect the accuracy of surveillance data. Among adult infectious disease studies, it has been shown that adhering to Infectious Disease Society of America guidelines for a variety of pathogenic processes leads to fewer complications, reduced mortality and infection recurrence, shorter hospital stays, lower readmission rates, and overall lower healthcare costs [21,22,23,24]. As local and national departments of public health continue to reassess case and surveillance definitions related to congenital syphilis, it may be advantageous to synchronize definitions and terminology that more closely reflect those used in clinical utilization practices.

Previous studies in other pediatric specialties, including those with various cardiac and pulmonary pathologic conditions, have demonstrated that consistent execution of guidelines in pediatric care has been shown to have positive medical outcomes [25,26]. However, these same studies demonstrated that there still exist temporal and regional variations in diagnostic and management practices, similar to our findings [25,26]. Another study looking at the transition of care from pediatric to adult specialty care, based on guideline recommendations in those with primary immunodeficiencies and autoinflammatory conditions, demonstrated improved treatment adherence, high-cost savings for healthcare institutions and patients, and long-term morbidity outcomes [27]. While previous studies saw variations in prenatal management in care among birthing parents who birthed infants born with congenital syphilis [3,28], there is a scarcity of literature investigating postnatal management differences. From the perspective of the provider, previous studies have shown that following national infectious disease guidelines can improve knowledge and reduce cognitive biases in clinical practice. It may reduce reliance on incorrect cognitive cues and allow for better evidence-based practices [29].

Our study has multiple limitations we wish to address. As with any large survey-based study, there is the potential for various types of bias [29,30]. There may be self-report bias due to social desirability or memory limitations. We attempted to overcome this by only including participants who directly or indirectly assist in the management of congenital syphilis. We attempted to overcome sampling bias by sampling from a nationwide pediatric provider database from all regions of the US [31], but we recognize these results may not be generalizable to certain areas within the US where response rates were lower, or outside of the US where responses were not in the inclusion criteria. There were a small number of participants who did not complete the survey in its entirety which may withhold valuable information, but this was a very small percentage of participants so did not have a significant impact on the power of the study. Additionally, while we attempted to make the questions as clear and understandable as possible, with the assistance of our institution’s Survey Laboratory [32], which has performed hundreds of validated survey studies to date across multiple specialties, there may still be instances where the wording and order of questions influenced respondents’ answers. Additionally, there were no financial or secondary incentives offered to potential participants, which may have further limited our ability to recruit and collect impactful data. 

The AAP has highlighted a critical shortage of pediatric medical subspecialists, including those in pediatric infectious diseases, in their workforce policy statement [33]. This shortage is compounded by geographic disparities, with many underserved areas lacking adequate access to pediatric infectious disease care [33]. A recently published study projects that the pediatric infectious disease workforce will grow more slowly than most other pediatric subspecialties from 2020 to 2040, exacerbating existing geographic disparities in access to care. Additionally, the number of fellows entering pediatric infectious disease training programs is low, with only about half of fellowship positions being filled, with salaries for pediatric infectious disease specialists among the lowest across medical subspecialties [34,35]. These factors collectively indicate a significant and ongoing shortage of pediatric infectious disease providers, which is expected to worsen unless addressed through targeted policy and training interventions. While there were only a few significant differences in practice between infectious disease and non-infectious disease providers in our study, it emphasizes the point that the diagnosis and management of congenital syphilis requires a multidisciplinary approach, and there may be geographic regions where general or neonatal pediatric providers more often manage cases of congenital syphilis without the assistance of a pediatric infectious disease subspecialist. 

Future directions include investigating congenital syphilis management strategies to identify factors that guide clinical decision-making and examining variations in patient outcomes associated with different clinical management approaches. Our findings may lead to additional studies that could investigate both short-term and long-term health consequences among infants with congenital syphilis with variable diagnosis and management practices. Additional future directions of this study include looking into financial implications of differences in practice and management, including cost-effective analyses of the various diagnostic workups, comparisons of health outcomes among those institutions that do or do not utilize certain laboratory tests and procedures, and more detailed investigation into the rise in costs of hospitalization for these patients who may have receive 10 days of penicillin therapy or 1 day of penicillin therapy in certain possible congenital syphilis scenarios. This study could also be expanded to include the clinical practices in countries outside of the US and compare outcomes with regard to the differences not only in diagnosis and management strategies of providers individually but also in different national and/or regional guidelines. 

As the incidence of congenital syphilis increases yearly, it is important to understand how different congenital syphilis presentations are handled in practice relative to available professional guidelines, as well as the sources of practice variability. This work serves as an important contribution toward defining the landscape of physician practices that shape clinical outcomes for newborns in the US and underscores the importance of clear and effective guidelines given the continued rise in identified congenital syphilis cases nationwide.

## Figures and Tables

**Table 1:** Demographic attributes of infectious disease (ID) and non-ID physicians.

	Total (*n* = 442) *n* (%)	ID (*n* = 153) *n* (%)	Non-ID (*n* = 289) *n* (%)	*p*-Value (χ^2^)
Gender				0.484
Man	182 (41.2)	62 (41)	120 (41.5)	
Woman	251 (56.8)	90 (59)	161 (55.7)	
Non-binary	1 (0)	0 (0)	1 (0)	
Prefers not to disclose	8 (2)	1 (1)	7 (2)	
Age (in years)				0.008
30–39	76 (17)	37 (24)	39 (14)	
40–49	106 (24.0)	42 (28)	64 (22)	
50–59	109 (24.7)	28 (18)	81 (28)	
60–69	113 (25.6)	32 (21)	81 (28)	
≥ 70	37 (8.4)	13 (9)	24 (8.3)	
Years since training completion				<0.001
0–5 years	64 (13)	59 (39)	132 (45.7)	
5–10 years	71 (15)	38 (25)	26 (9.0)	
10–15 years	52 (11)	15 (10)	36 (13)	
15–20 years	64 (13)	10 (7)	54 (19)	
>20 years	191 (39.7)	30 (20)	41 (14)	
Region of practice				0.013
Midwest	88 (20)	43 (28)	45 (16)	
Northeast	97 (22)	30 (20)	67 (23)	
South	175 (39.6)	51 (33)	124 (42.9)	
West	82 (19)	29 (19)	53 (18)	

**Table 2:** Diagnostic and treatment decisions: proven or highly probable congenital syphilis.

Diagnostic Testing	Total (*n* = 442) *n* (%)	ID (*n* = 153) *n* (%)	Non-ID (*n* = 289) *n* (%)	*p*-Value (χ^2^)
Lumbar puncture				0.002
Yes	434 (98.2)	146 (95.4)	288 (99.7)	
No	8 (2)	7 (5)	1 (0)	
Long-bone radiographs				0.003
Yes	420 (95.0)	139 (90.8)	281 (97.2)	
No	22 (5.0)	14 (9)	8 (3)	
Head imaging (US, CT, MRI)				0.288
Yes	210 (47.5)	78 (49)	132 (45.7)	
No	232 (52.5)	75 (51)	157 (54.3)	
Blood tests (CBC, LFTs)				0.963
Yes	439 (99.3)	152 (99.3)	287 (99.3)	
No	3 (1)	1 (1)	2 (1)	
Ophthalmologic examination				0.116
Yes	364 (82.4)	120 (78.4)	244 (84.4)	
No	78 (18)	33 (22)	45 (16)	
Treatment				0.162
Aqueous penicillin G for 10 days	404 (91.4)	146 (95.4)	258 (89.3)	
Procaine penicillin for 10 days	10 (2.3)	2 (1)	8 (3)	
Benzathine penicillin for 1 day	2 (0)	0 (0)	2 (1)	
Pending diagnostic results	26 (5.9)	5 (3)	21 (7.3)	

CBC = complete blood count with differential; CT = computer tomography; LFTs = liver function tests; MRI = magnetic resonance imaging; US = ultrasound.

**Table 3:** Diagnostic and treatment decisions: possible congenital syphilis.

Diagnostic Testing	Total (*n* = 440) *n* (%)	ID (*n* = 153) *n* (%)	Non-ID (*n* = 287) *n* (%)	*p*-Value (χ^2^)
Lumbar puncture				0.224
Yes	388 (88.2)	131 (85.6)	257 (89.6)	
No	52 (12)	22 (14)	30 (10)	
Long-bone radiographs				0.357
Yes	361 (82.1)	122 (79.7)	239 (83.3)	
No	79 (18)	31 (20)	48 (17)	
Head imaging (US, CT, MRI)				0.128
Yes	105 (21.6)	43 (28)	62 (22)	
No	335 (76.1)	110 (71.9)	225 (78.4)	
Blood tests (CBC, LFTs)				0.464
Yes	415 (94.3)	146 (95.4)	269 (93.7)	
No	25 (5.7)	7 (6)	18 (6.3)	
Ophthalmologic examination				0.550
Yes	207 (47.1)	69 (45)	138 (48.1)	
No	233 (52.9)	84 (55)	149 (51.9)	
No additional workup				
Yes	17 (3.9)	4 (3)	13 (4.5)	0.275
No	423 (96.1)	149 (97.4)	274 (95.5)	
Treatment				0.034
Aqueous penicillin G for 10 days	315 (71.3)	107 (69.9)	208 (72.0)	
Procaine penicillin for 10 days	7 (2)	1 (1)	6 (2)	
Benzathine penicillin for 1 day	38 (8.6)	8 (5)	30 (10)	
Pending diagnostic results	72 (1)	35 (23)	37 (13)	
No treatment	8 (2)	2 (1)	6 (2)	

CBC = complete blood count with differential; CT = computer tomography; LFTs = liver function tests; MRI = magnetic resonance imaging; US = ultrasound.

**Table 4:** Diagnostic and treatment decisions: congenital syphilis less likely.

Diagnostic Testing	Total (*n* = 421) *n* (%)	ID (*n* = 145) *n* (%)	Non-ID (*n* = 276) *n* (%)	*p*-Value (χ^2^)
Lumbar puncture				0.213
Yes	36 (8.6)	9 (6)	27 (9.8)	
No	385 (91.4)	136 (93.8)	249 (90.2)	
Long-bone radiographs				0.186
Yes	40 (11)	10 (6.9)	30 (11)	
No	381 (89.1)	135 (93.1)	246 (89.1)	
Head imaging (US, CT, MRI)				0.935
Yes	12 (2.9)	4 (3)	8 (3)	
No	409 (97.1)	141 (97.2)	268 (97.1)	
Blood tests (CBC, LFTs)				0.003
Yes	99 (24)	22 (15)	77 (28)	
No	322 (76.5)	123 (84.8)	199 (72.1)	
Ophthalmologic examination				0.467
Yes	22 (5.2)	6 (4)	16 (5.8)	
No	399 (94.8)	139 (95.9)	260 (94.2)	
No additional workup				0.008
Yes	319 (75.8)	121 (83.4)	198 (71.7)	
No	102 (24.2)	24 (17)	78 (28)	
Treatment	Total (*n* = 438)	ID (*n* = 153)	Non-ID (*n* = 285)	<0.001
Aqueous penicillin G for 10 days	14 (3.2)	4 (3)	10 (3.5)	
Procaine penicillin for 10 days	1 (0)	0 (0)	1 (0)	
Benzathine penicillin for 1 day	245 (55.9)	108 (70.6)	137 (48.1)	
Pending diagnostic results	16 (3.7)	5 (3)	11 (3.9)	
No treatment	162 (37.0)	36 (24)	126 (44.2)	

CBC = complete blood count with differential; CT = computer tomography; LFTs = liver function tests; MRI = magnetic resonance imaging; US = ultrasound.

## Data Availability

Data generated and analyzed during this study are included in their entirety within this report and its Supplementary Materials.

## Supplementary Materials

The following supporting information can be downloaded at https://www.mdpi.com/article/10.3390/jcm13206280/s1, Table S1: Summary of guideline recommendations for congenital syphilis (CS) evaluation and treatment in neonates born to mothers diagnosed with syphilis during pregnancy.

## Appendix A. Survey Instrument Questions

1. Do you currently directly or indirectly assist in the management of congenital syphilis for patients in North America?

(a) Yes

(b) No

2. What is your professional title/training?

(a) Pediatric Infectious Diseases (MD/DO)

(b) Pediatric Infectious Diseases (APN/PA/Advanced Practitioner)

(c) Pediatric Hospitalist (MD/DO)

(d) General Pediatrician (MD/DO)

(e) Neonatal Hospitalist/Neonatologist (MD/DO)

(f) I am not an MD/DO/APN/PA/Advanced Practitioner

(g) Other, please specify:

TEXT: __________________________________________________

- Please read the case description and pick the answers that most closely correspond to how you would handle such a case.
- An infant is born full term at 39 weeks gestation to a G1P1 28-year-old female. The mother was diagnosed with primary syphilis during the third trimester with an RPR of 1:8 and did not receive treatment. The infant is noted to have a diffuse maculopapular rash and hemorrhagic rhinitis. There are no central nervous system symptoms at this time. Infant RPR is 1:32
- You diagnose the patient with symptomatic/proven congenital syphilis.

3. Please select all additional diagnostics you would be likely to perform:

(a) Lumbar puncture with cerebrospinal fluid studies

(b) Long bone radiographs

(c) Head imaging including head ultrasound, CT scan or MRI

(d) Blood tests including complete blood count with differential, liver function tests

(e) Ophthalmologic examination

(f) No additional work up

4. Which treatment would you prescribe?

(a) Aqueous penicillin G for 10 days

(b) Procaine Penicillin for 10 days

(c) Benzathine Penicillin for 1 day

(d) No treatment

(e) I would not make this decision without the result of lumbar puncture and/or bone radiographs

- Please read the case description and pick the answers that most closely correspond to how you would handle such a case.
- An infant is born full term at 39 weeks gestation to a G1P1 28-year-old female. The mother was diagnosed with primary syphilis during the third trimester with an RPR of 1:8. She received x1 dose of IM Benzathine penicillin 1 week prior to delivery. The infant is noted to be asymptomatic at birth. Infant RPR is 1:2.
- You diagnose the baby with possible congenital syphilis.

5. Please select all additional diagnostics you would be likely to perform:

(a) Lumbar puncture with cerebrospinal fluid studies

(b) Long bone radiographs

(c) Head imaging including head ultrasound, CT scan or MRI

(d) Blood tests including complete blood count with differential, liver function tests

(e) Ophthalmologic examination

(f) No additional work up

6. What treatment would you prescribe?

(a) Aqueous penicillin G for 10 days

(b) Procaine Penicillin for 10 days

(c) Benzathine Penicillin for 1 day

(d) No treatment

(e) I would not make this decision without the result of lumbar puncture and/or bone radiographs

- Please read the case description and pick the answers that most closely correspond to how you would handle such a case.
- An infant is born full term at 39 weeks gestation to a G1P1 28-year-old female. The mother was diagnosed with primary syphilis during the third trimester with an RPR of 1:8. She received x1 dose of IM Benzathine penicillin 1 week prior to delivery. The infant is noted to be asymptomatic at birth. Infant RPR is 1:2.
- You diagnose the baby with possible congenital syphilis.

7. Please select all additional diagnostics you would be likely to perform:

(a) Lumbar puncture with cerebrospinal fluid studies

(b) Long bone radiographs

(c) Head imaging including head ultrasound, CT scan or MRI

(d) Blood tests including complete blood count with differential, liver function tests

(e) Ophthalmologic examination

(f) No additional work up

8. What treatment would you prescribe?

(a) Aqueous penicillin G for 10 days

(b) Procaine Penicillin for 10 days

(c) Benzathine Penicillin for 1 day

(d) No treatment

(e) I would not make this decision without the result of lumbar puncture and/or bone radiographs

9. Demographics
9.1. What is your age?

(a) 20–29 years old

(b) 30–39 years old

(c) 40–49 years old

(d) 50–59 years old

(e) 60–69 years old

(f) 70+ years old

9.2 Do you identify as:

(a) A man

(b) A woman

(c) Non-binary

(d) A different gender identity

(e) Prefer not to disclose

9.3 Since completing your highest training level, how long have you been practicing?

(a) 0–5 years

(b) 5–10 years

(c) 10–15 years

(d) 15–20 years

(e) >20 years

9.4 In what region of the United States do you practice?

(a) Midwest

(b) Northeast

(c) South

(d) West

(e) Canada

(f) Outside of the US or Canada
