# Supplementary material for: Evaluation and Treatment of Congenital Syphilis: A National Survey of US Pediatric Specialists
Source: J Clin Med. 2024 Oct 21;13(20):6280. doi: 10.3390/jcm13206280 (PMC11508399; doi:10.3390/jcm13206280)
Supplement: Supplementary file 1 [file jcm-13-06280-s001.zip › Supplemental Table S1.pdf]

Supplemental Table S1: Summary of Guideline Recommendations for Congenital Syphilis (CS) Evaluation and Treatment in Neonates Born to Mothers Diagnosed with Syphilis During Pregnancy

Presentation	Designation	Recommended Evaluation	Recommended Treatment
Any of the following:<br>▪ Abnormal physical exam, findings consistent with CS<br>▪ Serum nontreponemal serological titer ≥ 4X maternal titer at delivery<br>▪ Positive PCR or darkfield, silver stain of body fluids, tissue	Proven or highly probable CS	▪ **Lumbar puncture**<br>▪ **CBC with differential**<br>▪ ***Long bone radiographs ****<br><br>+/- chest radiograph, liver function tests, neuroimaging, ophthalmologic exam, auditory brain stem response as clinically indicated	**Preferred:** Aqueous penicillin G for 10 days<br>Alternative: Procaine penicillin G for 10 days
Normal physical exam, serum nontreponemal titer ≤ 4X maternal titer at delivery, and any one of the following:<br>▪ Absent, inadequate, or undocumented maternal treatment<br>▪ Non-recommended maternal treatment (i.e. nonpenicillin agent)<br>▪ Recommended maternal treatment initiated < 30 days before delivery	Possible CS	▪ **Lumbar puncture ****<br>▪ **CBC with differential ****<br>▪ **Long bone radiographs ****	**Preferred:** Aqueous penicillin G for 10 days<br>Alternative: Procaine penicillin G for 10 days<br><br>*Single-dose benzathine penicillin G acceptable if all recommended evaluation studies complete and normal.*
Normal physical exam, serum nontreponemal titer ≤ 4X maternal titer at delivery, and both of the following:<br>▪ Recommended maternal treatment initiated ≥ 30 days before delivery<br>▪ No evidence of maternal reinfection, relapse	CS less likely	None	**Preferred:** Single-dose benzathine penicillin G<br>Alternative: Re-check titer every 2-3 months until nonreactive without penicillin therapy if maternal titers decreased ≥ 4X or stable at low titer after treatment.

* US Centers for Disease Control and Prevention (CDC) guidelines recommend long bone radiography for all cases of proven or highly probable CS. American Academy of Pediatrics (AAP) guidelines recommend long bone radiography when clinically indicated.

** CDC guidelines specify evaluation studies can be foregone if 10 days of parenteral penicillin therapy completed.

CBC, complete blood count; PCR, polymerase chain reaction
